# A biological network-based regularized artificial neural network model for robust phenotype prediction from gene expression data

**DOI:** 10.1186/s12859-017-1984-2

**Published:** 2017-12-19

**Authors:** Tianyu Kang, Wei Ding, Luoyan Zhang, Daniel Ziemek, Kourosh Zarringhalam

**Affiliations:** 1 Department of Computer Science, University of Massachusetts Boston, 100 Morrissey Boulevard, Boston, 02125 MA USA; 2 Inflammation and Immunology, Pfizer Worldwide Research & Development, Berlin, Germany; 3 Department of Mathematics, University of Massachusetts Boston, 100 Morrissey Boulevard, Boston, 0212 MA USA

**Keywords:** Artificial neural network, Gene regulatory networks, Prediction of response, Clinical trial, Group Lasso

## Abstract

**Background:**

Stratification of patient subpopulations that respond favorably to treatment or experience and adverse reaction is an essential step toward development of new personalized therapies and diagnostics. It is currently feasible to generate omic-scale biological measurements for all patients in a study, providing an opportunity for machine learning models to identify molecular markers for disease diagnosis and progression. However, the high variability of genetic background in human populations hampers the reproducibility of omic-scale markers. In this paper, we develop a biological network-based regularized artificial neural network model for prediction of phenotype from transcriptomic measurements in clinical trials. To improve model sparsity and the overall reproducibility of the model, we incorporate regularization for simultaneous shrinkage of gene sets based on active upstream regulatory mechanisms into the model.

**Results:**

We benchmark our method against various regression, support vector machines and artificial neural network models and demonstrate the ability of our method in predicting the clinical outcomes using clinical trial data on acute rejection in kidney transplantation and response to Infliximab in ulcerative colitis. We show that integration of prior biological knowledge into the classification as developed in this paper, significantly improves the robustness and generalizability of predictions to independent datasets. We provide a Java code of our algorithm along with a parsed version of the STRING DB database.

**Conclusion:**

In summary, we present a method for prediction of clinical phenotypes using baseline genome-wide expression data that makes use of prior biological knowledge on gene-regulatory interactions in order to increase robustness and reproducibility of omic-scale markers. The integrated group-wise regularization methods increases the interpretability of biological signatures and gives stable performance estimates across independent test sets.

## Background

One of the main challenges of precision medicine is to identify patient subpopulation based on risk factors, response to treatment and disease progression. Our current inability in identifying disease specific and reproducible biomarkers has significantly contributed to the rising cost of the healthcare expenditure. There is a critical need for development of novel methodologies for patient stratification based on specific risk factors. To this end, large scale biological data sets such as genomic variations [1–3], transcriptomics [4–7] and proteomics [8, 9] have been extensively used to derive prognostic and diagnostic biomarkers for specific diseases. Although these models have had relative success in specific areas, particularly in the field of oncology [10], their overall reproducibility is a major concern [11–15]. One of the main reasons for this apparent lack of reproducibility is the high degree of genetic heterogeneity in human populations. Other contributing factors include low sample sizes and high dimension of the measured feature spaces, which make classification algorithms prone to ‘overfitting’ [15–18]. Several models have been developed by the research community to address these challenges. In particular, regularization models are very popular in addressing the high dimension of biological datasets [19–21]. Although these methods generally have acceptable performance in cross validation studies, their reproducibility in independent datasets is not typically assessed [22].

Over the past few years, there has been a growing interest in approaches that integrate information on molecular interactions, such as canonical pathways, GO annotation or protein-protein interactions into biomarker discovery and response prediction algorithms. Indeed, novel approaches for leveraging prior biological knowledge for biomarker discovery are emerging as a promising alternative to data-driven methods [17, 23–30]. For instance, authors in [31, 32] propose regression models with a graph-based penalty to impose similar weights to genes that are closer together in a given network. There are several types of networks that encode prior biological knowledge on biomolecular interactions. Information on gene regulatory interactions in particular, can be effectively used to address the high dimensionality of the data sets. Gene regulatory networks provide a way to identify active regulatory mechanisms and their potential association to the phenotype. Leveraging such information into the classification or regression tasks can result in more optimal sparsity and identification of reproducible markers.

In this work, we develop a Regularized Artificial Neural Network (ANN) that encodes the co-dependencies between genes and their regulators into the architecture of the classifier. Our model, GRRANN (**G**ene **R**egulatory network-based **R**egularized **A**rtificial **N**eural **N**etwork), is specifically designed for prediction of phenotypes from gene-expression data. The induced sparsity on the ANN based on the gene-regulatory interactions, significantly reduces the number of model parameter and the need for large sample sizes that are typically required to train ANNs. The structure of our ANNs naturally lends itself to regularization models for group-wise and graph-based variable selection. In particular, group-wise regularization of gene-sets based on their regulatory interactions can be achieved with relative ease using our model. Group-wise shrinkage of covariates has been extensively studied in the framework of penalized linear and logistic regression [33–36]. This penalty is particularly useful for transcriptomics data, where co-regulated gene sets are present in abundance. However, the group-wise regularization as originally proposed, exhibits undesirable effects in the regression task when there is overlap between groups of covariates, which is almost always the case in co-regulated gene sets [35]. Generalizations of this penalty have been proposed to overcome this difficulty [36]. Nevertheless, calculating the generalized penalty can be computationally expensive. We will show that all of these limitations are naturally avoided in our ANN design. In addition to group-based penalties, we will enforce single gene based regularity conditions in our fitting process.

We focus our study on human clinical trials with the goal of identifying responders to treatment using the baseline or early treatment gene expression data. Importantly, in addition to cross validation studies, we will demonstrate the generalizability of our method using truly independent test sets. We used the following criteria for selecting independent train and test sets: (1) a dataset of at least 20 human subjects with a defined clinical binary outcome, i.e. responders and non-responders, (2) at least some detectable difference in gene expression at baseline between the two groups, and (3) the availability of a similar but entirely independent trial for testing purposes. For the purposes of this work, we settled on two datasets: the studies in [37, 38] on acute rejection in kidney transplantation as well as the the study on the infliximab treatment of ulcerative colitis in [39].

For the choice of the network, we rely on causal/non-causal protein-protein and protein-gene interactions in the STRING DB database [40]. This network consists of approximately ∼40,000 nodes and ∼400,000 edges. The released package comes with version 10 of the STRING DB database.

## Methods

**Fig. 1:**
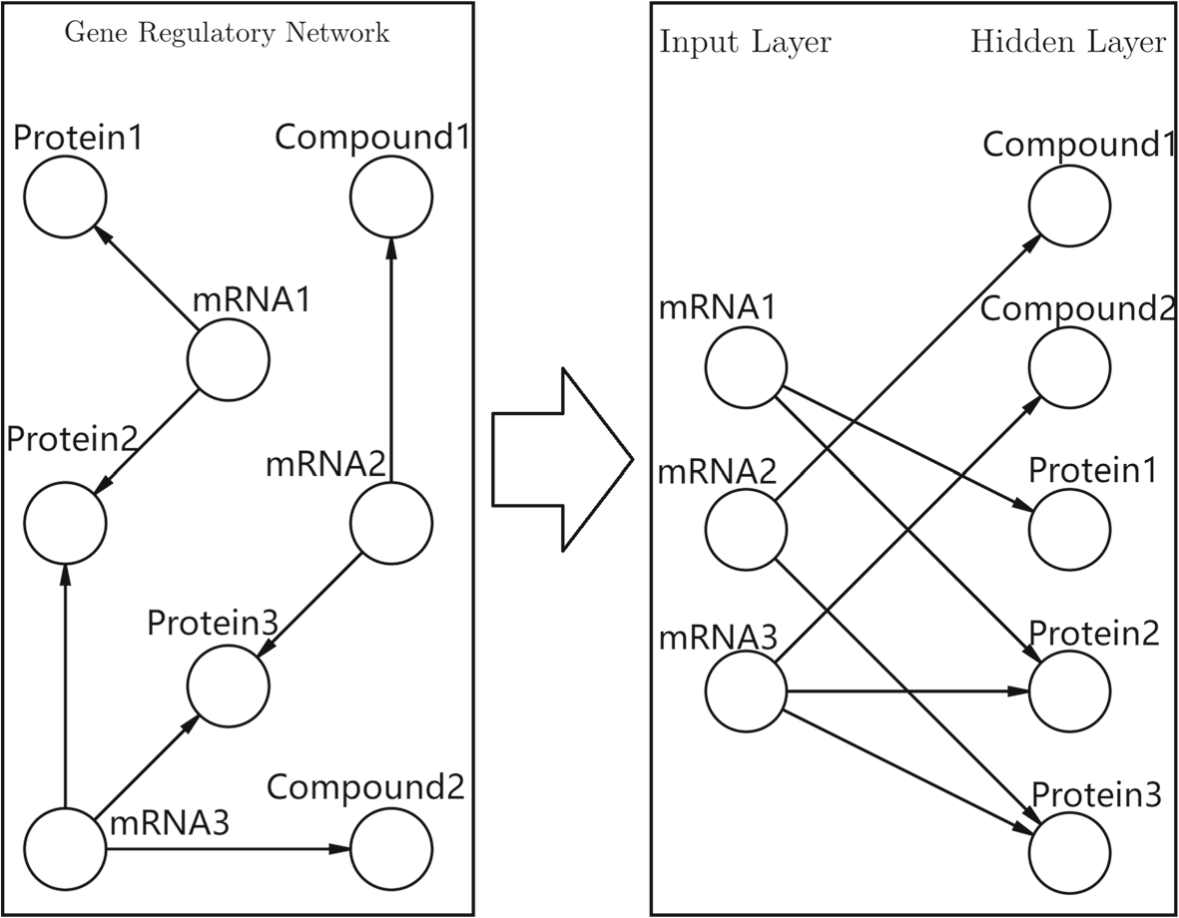
Figure illustrates the conversion of a gene regulator network (GRN) into an artificial neural network (ANN). The left panel shows regulatory interactions between genes and their upstream regulators (e.g., Proteins, Compounds, etc.). The panel on the right side represents the input and the hidden layer of the induced ANN based on the gene regulatory interactions. Each mRNA-regulator interaction in the GRN correspond to a input-hidden node connection in the ANN

**Fig. 2:**
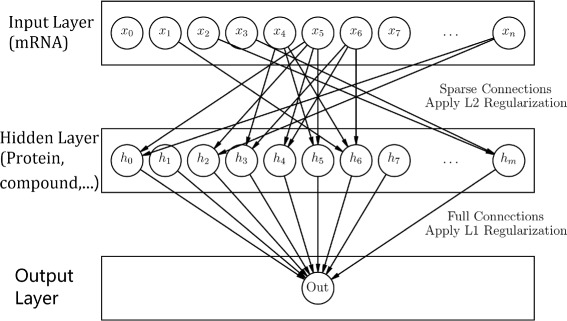
Figure represents a gene regulatory network based ANN. The input layer corresponds to genes, while the hidden layer correspond to regulators. The connections between the input and the hidden layer are based on regulatory interactions. The ridge *ℓ*_2_ regularization is applied on these connection. The output layer consists of a single node for binary classification. The nodes in hidden layer are fully connected to the output node. The *ℓ*_1_ regularization is applied to these connections

Our goal is to develop a neural network classifier for predicting phenotypes (e.g., response to therapy) from baseline gene expression data in a manner that incorporates information on gene regulatory interactions in the design of the network. The intuition is that taking interaction between genes and regulatory mechanisms into consideration should result in optimal model sparsity, which helps in avoiding overfitting. To this end, we design a gene regulatory network based artificial neural neural network model together with regularization methods for simultaneous shrinkage of gene-sets based on ‘active’ upstream regulatory mechanisms. The starting point of our method is a network of gene regulatory interactions of the type, ‘regulator *r* upregulates gene *g*’ or ‘regulator *r* downregulates gene *g*’. We encode this information in a (signed) graph *G* consisting of nodes *V* and a set of edges *E*. The regulatory nodes are typically proteins, miRNAs, compounds, etc., and the terminal nodes are mRNAs. The edges in *E* indicate a regulatory interaction between a source node (regulator) and a target node (gene). When the direction of the regulation is known, the edge will have a sign with + indicating upregulation and - indicating downregulation. From this regulatory network, we construct an ANN as follows. The ANN consists of an input layer, a single hidden layer and one output layer. The nodes in the input layer correspond to genes, while the nodes in the hidden layer correspond to the regulators in the network. The connections from the input layer to the hidden layer are based on the gene regulatory network, i.e., an input node is connected to a hidden node if and only if the corresponding regulatory interaction exists. Figure 1 shows the construction of the input and the hidden layers from the gene regulatory network. The output layer consists of a single node for binary classification. Every node in the hidden layer is connected to the output node. This design results in a sparse ANN with significantly fewer edges than a fully connected ANN. As such, fitting the parameters of this ANN will require significantly less amount of data. Figure 2 shows a schematic representation of the ANN.

We may consider alternative architectures as well. For instance, we can construct networks from edges of a specific type only (+ or −). Given a set of training data $\{(y_{i},x_{i})\}^{n}_{i=1}$, with $x_{i}\in \mathbb {R}^{p}$ representing a vector of normalized gene expression values and *y*_*i*_∈{0,1} representing a binary response, we would like to solve the following optimization problem

1 $$ \underset{W}{\text{argmin}} \frac{1}{n}\sum\limits_{i=1}^{n}\Phi_{W}(y_{i},x_{i}) + g(\alpha, \lambda, W)  $$

where *Φ*_*W*_ is the ANN loss function, *W* represent the matrices of parameters (weights) of the ANN, *g*(*α*,*λ*,*W*) is a penalty term, and *α* and *λ* are tuning parameter. The parameter *W*=(*W* ^(1)^,*W* ^(2)^) of the ANN, corresponding to weights between the input and the hidden layer, *W* ^(1)^, and the weights between the hidden layer and the output layer, *W* ^(2)^. In our model, the loss (error) function is set to the cross entropy (log likelihood) function:

2 $$ \Phi_{W}(y_{i},x_{i}) = y_{i} \log(\hat{y}_{i}) + (1-y_{i})log(1-\hat{y}_{i})  $$

where $\hat {y}_{i} = f_{2}(W^{(2)}f_{1}(W^{(1)}x_{i} + b^{(1)}) + b^{(2)})$ is the output of the ANN. Here, *f*_1_ and *f*_2_ are activation functions that are applied point-wise and *b* ^(1)^ and *b* ^(2)^ are bias terms. For activation function of the ANN, we utilized the rectified linear function (ReLU), *f*_1_(*x*)=*max*(0,*x*), for the hidden layer and the sigmoid function *f*_2_ for the output layer. The ReLU is selected due to its advantage in avoiding the problem of vanishing gradient.

### Regularization

Let $W_{ij}^{(1)}$ denote the weight of the edge from the *j*-th gene to the *i*-th regulator and let $W_{i}^{(2)}$ denote the weight of the edge from the *i*-th regulator to the output layer. The gene regulatory network and correspondingly the ANN, group the genes into (overlapping) gene-sets according to the upstream regulatory mechanisms (hidden nodes of the ANN). We would like to introduce simultaneous shrinkage of these gene-sets through the penalty term *g*(*α*,*λ*,*W*). This can be achieved by imposing an *ℓ*_1_ penalty of the form ||*W* ^(2)^||_1_ in the optimization problem 2. This penalty, is the so called ‘group-lasso’ penalty in regression models [35].

In situations where the true underlying mechanism of the phenotypic difference between patient groups is governed by differential regulatory elements, it would be advantageous to eliminate gene-sets that correspond to inactive regulatory mechanisms. Recall that the nodes in the hidden layer of the ANN correspond to the regulators. Hence, regularizing nodes in this layer, will correspond to selection of gene-set based on active regulatory mechanism. Note that some genes may participate in multiple regulatory interactions and should be eliminated due to inactive interactions only. This is the main reason for the introduction of the ‘overlap’ group-lasso in regression [36]. However, in our formulation, there is no need for such costly considerations. Once a particular weight $W_{i}^{(2)}$ is set to 0, the weight of the genes connecting to the *i*-th regulator, i.e., $W_{ij}^{(1)}$ will no longer enter the fitting process and will be dropped out. Genes corresponding to the dropped out edges can still influence the output through weights that correspond to other active hidden nodes. Weight scaling can also be introduced for differential shrinkage of the hidden nodes based on the number of incoming connections. Additionally, an *ℓ*_2_ penalty term on *W* ^(1)^ can be added to the model for elastic net effects [41]. Note that co-regulated genes tend to have correlated expression. The addition of the *ℓ*_2_ penalty will have the effect of assigning similar weights to such genes. Alternatively, the *ℓ*_2_ penalty on *W* ^(1)^ can be replaced with an *ℓ*_1_ penalty for within group sparsity. The full penalty function is then

3 $$ g(\alpha, \lambda, W) = \alpha \lambda ||W^{(1)}||_{2} + (1 - \alpha) \lambda \sum\limits_{i} \sqrt{\rho_{i}} |W_{i}^{(2)}|  $$

where *ρ*_*i*_’s are the number of incoming edges for the *i*-th hidden node and *α*∈[0,1] is tradeoff factor.

The tuning parameter *λ* is set by a search strategy as follows. For a very large value of *λ*=*λ*_*max*_, the *ℓ*_1_ penalty will set all the weights to zero. We obtain an appropriately large *λ* value by trial and error. We then set *λ*_*min*_=0.1*λ*_*max*_ and assess the performance of the model for a grid of *λ* values between *λ*_*min*_ and *λ*_*max*_ and record the best performing *λ*.

### Data sets and preprocessing

We processed gene expression data from two clinical phenotypes; (1) acute rejection in kidney transplantation [37, 38] and (2) response to infliximab in ulcerative colitis [39]. Each phenotype consists of two datasets (GEO accession numbers GSE50058 and GSE21374 in acute rejection and GSE12251 and GSE14580 in response to infliximab).

The dataset GSE50058 consists of 43 kidney transplant rejection and 54 non-rejection samples. Dataset GSE21347 consists of 76 kidney transplant rejection and 206 non-rejection samples.

The datasets GSE14580 consists of 24 patients with active ulcerative colitis. Patients were treated with 5 mg/kg infliximab and response was assessed at week 4 or 6 after infliximab treatment. There are a total number of 8 responders and 16 non-responders in this dataset. Dataset GSE12251 consists of 22 patients with active ulcerative colitis. Patients are treated with 5 mg/kg or 10 mg/kg infliximab and response was assessed at week 8 after infliximab treatment. There are a total of 12 responders and 10 non-responders in this dataset.

Datasets corresponding to different phenotypes were analyzed separately. For each phenotype, datasets were RMA (Robust Multi-array Average) normalized. Probes that were absent in all samples - irrespective of response status - were filtered using the mas5calls function from the R Bioconductor package [42]. In addition, each dataset was standardized by subtracting column means and dividing by standard deviations prior to training. Genes that were not present in the network of regulatory interactions were filtered out. Training and testing data sets were separately standardized to mean 0 and standard deviation 1.

### Assessing model performance

**Fig. 3:**
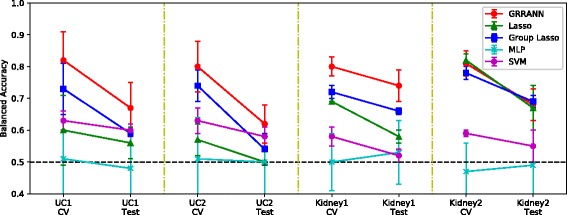
Overview of model performance in terms of balanced accuracy in cross-validation (labeled as ‘CV’) and independent test sets (labeled as ‘Test’). Black dash line indicate random performance. Each category (Kidney and UC) consist of two independent clinical trial datasets. In each panel, the left end points indicate the model performance in CV trained on the indicated training set and the right endpoints indicate the performance in independent test set. A 5-fold cross validation was utilized in all experiments. The red line segments indicate the performance of our model GRRANN. Alternative models are group lasso (blue), *ell*_1_ regularized logistic regression (green), a multilayer perceptron (cyan) and a support vector machine (purple)

**Fig. 4:**
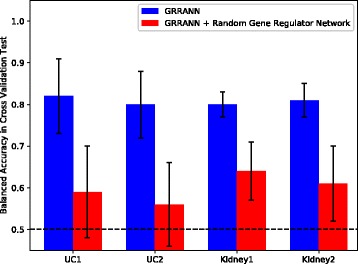
Figure depicts average cross validation results in multiple runs of GRRANN (blue) and a randomized version of the model (red), where connections between the hidden and the input nodes are fully shuffled. As can be seen, correct regulatory connections have a significant impact on model performance. Same regularization settings were utilized in both tests

**Fig. 5:**
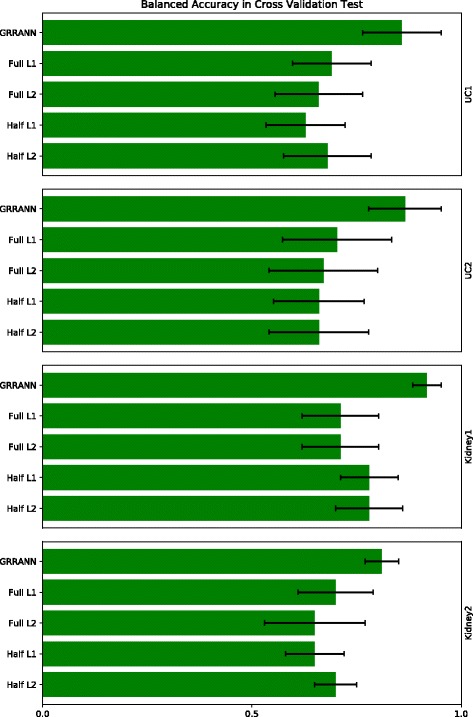
Figure shows the impact of the choice of penalty on model performance. The bar plots indicate the average cross-validation balanced accuracy in multiple runs. In all experiments a regularization of the form *r*_1_−*r*_2_ has been applied where *r*_1_ indicated the regularization applied to the weights in the first layer *W* ^(1)^ and *r*_2_ indicates the regularization applied to the weights in the second layer *W* ^(2)^. Half L2: *ℓ*_2_-*Null*), Half L1: *Null*- *ℓ*_1_, Full L1: *ℓ*_1_- *ℓ*_1_, Full L2: *ℓ*_2_- *ℓ*_2_ and GRRANN: *ℓ*_2_- *ℓ*_1_

**Fig. 6:**
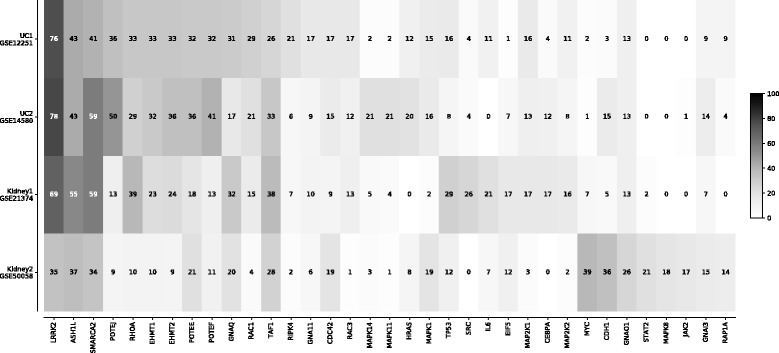
Figure shows a heatmap of the number of times that regulators appear in the top 10 in the list of nodes ranked by the magnitude of the weights in each bootstrap run

The performance of all models were assessed using cross validation as well as independent train and test sets. We benchmarked our method GRRANN (Gene Regulatory Network-based Regularized Artificial Neural Network) against several other ANN designs, penalized regression models and SVMs. The benchmarks were specifically selected to test various aspects of our model and can be divided into three categories. First, to test the importance of the topology of the gene regulatory network, we compared the performance of our model against other ANN designs including a) a fully connected ANN with two hidden layers, each containing 20 neurons and b) a randomized version of our ANN, where number of layers, nodes and connections are identical but the connections between the input and the hidden layer are randomized. The second class of experiments were performed to assess the effect of regularization on our ANN. These models are identical in structure and the only difference is in the type of the enforced regularization. They are a) no group regularization, corresponding to *α*=1, b) no ridge regularization, corresponding to *α*=0. Additionally we tested the effect of interchanging *ℓ*_1_ and *ℓ*_2_ norms in both layers for a fixed *α*=0.5. More specifically, we tested c) replacing ridge penalty on *W* ^(1)^ with lasso and d) replacing group lasso on *W* ^(2)^ with group ridge. The third category of benchmarks were performed to compare our method with other alternative state-of-the-art classifiers, including 1) regularized logistic regression models of elastic nets and 2) sparse group lasso and c) a support vector machine with an RBF kernel. The benchmarks were performed using cross-validation as well as train and test on independent sets. Importantly, the independent test were performed to track model robustness to overfitting. Train and test sets were from completely independent, but similar clinical trial studies of the same disease (see section Data sets and preprocessing). Figures 3, 4, 5 and 6 summarize the results.

### Assessing robustness of predictions

To assess the consistency of activated neurons in predicting response, we implemented a bootstrap approach for tracking robustness against variations in training data. More specifically, the training data was sampled with replacement to generate 100 new training sets. The ANN was then trained on each bootstrap sample independently and the magnitude of the weights from the hidden units to the output unit were recorded. The hidden nodes were then ranked according to the magnitude of their weights to obtain a total of 100 ranked lists. We then tracked the number of times that the hidden units appeared on top of the lists (top 10). Robust predictors were then identified as those that consistently ranked high. Consistency was determined by examining the distribution of frequencies and selecting hidden units on the upper quantiles. This analysis may also facilitate and enhance the interpretability of the results. Since the hidden nodes in the ANN correspond to regulators in the gene regulatory network, an active hidden node with a high weight may thus indicate that the corresponding regulatory mechanism and its downstream genes associate significantly with the phenotype.

## Results

In this section, we present the cross-validation and independent test results for various benchmarks as mentioned in Methods. There are a total of 4 data sets in two groups; a) the acute kidney rejection dataset consisting of independent clinical trial data GSE21374 (Kidney1) and GSE50058 (Kidney2) and b) response to Infliximab in ulcerative colitis patients consisting of independent clinical trial data GSE12251 (UC1) and GSE14580 (UC2). Cross validations were performed independently on each of the 4 datasets using a 5-fold cross validation procedure. For independent train and test, the models were trained on one of the clinical trial data in a category (kidney or UC) and performance was assessed using the other data in the same category.

Figure 3 shows an overview of performance in terms of balanced accuracy split by cross-validation and independent test set runs. Random performance is indicated by the horizontal black lines. The main point of this benchmark is to test a) the performance against other state-of-the-art methods and b) track the consistency of the model in CV vs. independent tests. In every experiment, our method GRRANN consistently demonstrates equivalent or better performance than all other models. Other methods include *ℓ*_1_ regularized logistic regression (lasso), selected as a representative of gene-based regularized models, group-lasso selected as a representative of group-wise shrinkage models a fully connected multi layer perception (MLP) with 2 hidden layers with 20 neurons in each as a representative of non-regularized ANN models and a support vector machine(SVM) with RBF kernel. Notably the MLP model performance is random, indicating the importance of regularization in controlling overfitting and dimension reduction. The performance of the SVM is also suboptimal, likely due to overfitting. Lasso on the other hand, performs reasonably well in cross validation in Kidney rejection where sample numbers are high, however it fails to generalize to independent tests, indicating the importance of network-based regularization. Moreover, in UC data where the sample numbers are low, lasso performs poorly. This suggests that covariate-based regularization can not adequately handle high dimensional datasets. This also demonstrates the advantage of leveraging prior biological knowledge in reducing the dimension of omic-scale datasets. Group-lasso uses the same prior biological knowledge as our method. Gene sets are defined according to their upstream regulators using the same gene regulatory network as in our model. The gene sets are then penalized using a group-lasso penalty, corresponding to regularization of the weights in the second layer in our model. As can be seen group-lasso performs well in the kidney data set and the performance does not deteriorate significantly, indicating the relevance of gene regulatory mechanism in identifying reproducible markers of the disease. The behavior of group lasso is similar to our model, however, our model outperforms group lasso in all experiments, demonstrating the advantage of ANN designs over logistic regression models. Finally, the average decrease in balanced accuracy of our model between cross validation and independent train and test is about 16.0*%* across all samples. This is reasonable drop in accuracy given that the training and testing sets are completely independent clinical trial data.

Next, we sought to assess the significance of the gene-regulatory interactions on the performance of the model. To test this, we randomized the connections between the input and the hidden layer. More precisely, in these experiments we keep the nodes in the input and the hidden layers fixed, but shuffle the connections between them randomly. We utilized the same regularization in the randomized version as in the original case. Figure 4 shows the results of this experiment in terms of balanced accuracy in cross validation. As can be seen, shuffling the edges significantly deteriorates the performance of the model. This result, strongly indicates the importance of the true gene regulatory interactions in identifying markers of the disease. Additionally, we examined the weights of the fitted randomized model and noticed that the edges with high weights exist in the real network as well (i.e., the shuffling did not change the connection), indicating that real connections will increase the performance of the model.

The next set of benchmarks were designed to test the impact of alternative regularizations. As discussed earlier, we apply *ℓ*_1_ regularization to the weights of the second layer and an additional *ℓ*_2_ regularization to the weights of the first layer. The intuition behind the choice of *ℓ*_1_ penalty for the second layer is that this regularization eliminates inactive regulatory mechanisms and their down-stream genes. As such only genes participating in differentially expressed regulatory mechanisms between the two groups should enter the model. This is particularly advantageous in cases where the underlying difference between the two patient groups is governed by upstream regulators of differentially expressed genes. As for the *ℓ*_2_ part, the intuition is that genes under regulation of the same active regulators tend to have correlated expression. The ridge *ℓ*_2_ regularization is particularly useful in pulling correlated covariates close to one another by assigning similar weights and hence reducing model variance.

As discussed in “Methods” section, we replaced these regularization with alternative methods including a) deactivating group regularization (experiment labeled ‘Half L2’), b) deactivating ridge regularization (experiment labeled ‘Half L1’), c) replacing ridge penalty with lasso (experiment labeled ‘Full L1’) and d) replacing group lasso with group ridge (experiment labeled ‘Full L2’). In the latter 2 experiments the parameter *α* is set to 0.5 as in our mixed *ℓ*_2_- *ℓ*_1_ model. The network structure is identical in all these models. Figure 5 shows the average accuracy in cross validation. As can be seen, the proposed model of mixed *ℓ*_2_- *ℓ*_1_ outperforms all other combinations, confirming the intuition behind our choices.

Finally we performed a bootstrap study to investigate robustness of regulatory nodes to variations in datasets. More specifically, we performed a bootstrap analysis by training and cross validating the models using 100 random samples of each dataset and tracking the frequency of the selected predictors. Figure 6 shows a heatmap of the frequencies of top ranked hidden units in each dataset.

### Biological interpretation of the results

**Fig. 7:**
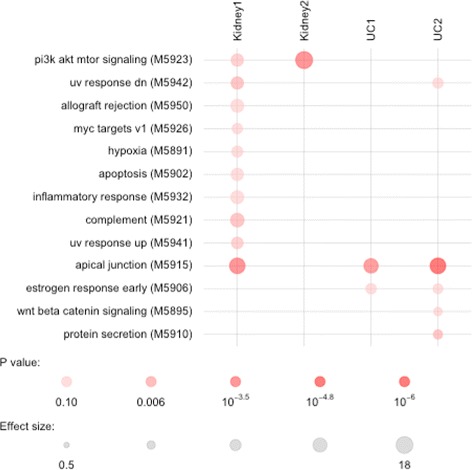
Figure shows the results of an enrichment analysis for all protein nodes that have been identified at least once in our 100 resampling runs

We examined the biological plausibility of the robust regulators, i.e., consistently activated hidden neurons. These hidden neurons already represent aggregation of underlying transcripts. As is apparent from Fig. 6, several protein nodes occur frequently but are not specific to any one dataset. In several cases, they appear to aggregate general immune system-related transcripts and are important for discriminatory power in all 4 datasets tested here. LRRK2, the most frequently associated hidden node across the datasets, has indeed been associated with inflammatory bowel disease [43] as well as kidney injury [44]. Figure 7 shows the results of an enrichment analysis for all protein nodes that have been identified at least once in our 100 resampling runs. For this analysis, we used the TMOD R package with a standard hypergeometric test [45] and a false discovery threshold of 0.1. The underlying gene set database is the hallmark subset of the MSIGDB collection [46] that has been specifically generated to reflect well-defined biological states and processes. In this analysis, distinct patterns become more apparent. The *allograft rejection* gene set is appropriately enriched in the Kidney1 dataset that contains expression data from renal allograft biopsies. A strong driver of this signal is the well-known cytokine IL6 which has been associated with allograft rejection previously [47]. IL6 is also picked frequently in the Kidney2 dataset, though overall the *allograft rejection* gene set does not reach significance in that dataset. The *PI3K/AKT/MTOR* shows the strongest enrichment shared by the two kidney rejection datasets. Indeed, this pathway has been discussed in the literature as related to renal transplant rejection [48]. Furthermore, Rapamycin, the prototypical inhibitor of MTOR, is FDA-approved for immune suppression after transplant surgery. The *apical junction complex* set is a highly plausible enrichment for the ulcerative colitis datasets as this complex regulates the intestinal barrier compromised in inflammatory bowel disease [49]. Taken together, these results in conjunction with previous benchmarks indicate that our model can accurately predict response in a consistent manner.

## Discussion and conclusion

In this paper we developed an regularized gene regulatory network-based artificial neural network classifier for predicting phenotypes from transcriptomics data in clinical trials. The design of the ANN architecture is based on the regulatory interactions between genes and their upstream regulators as encoded in a gene regulatory network were the hidden units and their connections to the input units in the ANN correspond to gene regulators and their downstream genes. The induced sparsity in the connections in our design significantly helps in avoid overfitting and the need for large amount of training samples, which is a drawback of conventional ANNs. The requirement for large training samples is particularly problematic in clinical studies, where the number of measurements is orders of magnitude larger than the number of samples. The incorporated regularizations as implemented in our model, penalize gene-sets based on the relevance of their upstream regulators to the phenotype. Additional penalties for elastic net effect, where co-regulated genes are assigned similar weights, are also integrated into the model, resulting in low model variance across datasets. In a series of benchmarks, we demonstrated that our model is able to identify reproducible and predictive signatures of response. Our benchmarks indicate that in training classifiers on high dimensional transcriptomics datasets, the model may still overfit and result in poor generalization to independent tests. By integrating prior knowledge into the classification framework the model will be more likely to select predictors that are more biologically relevant.

We provide the java code of our method along with a parsed version of the STRING DB network and the datasets used in this work. To increase the usability of our package, we provide pre-built java files as well as a graphical user interface. The package is available for download at https://github.com/kangtianyu/GRRANN. As future work we plan to investigate theoretical properties of the regularization parameter *λ* and alternative structures and regularizations that can further reduce the need for large training samples.

## Abbreviations

ANN: Artificial neural network
CV: Cross validation
GRRANN: Gene regulatory network-based regularized artificial neural network
GRN: Gene regulator network
MLP: Multi layer perception
ReLU: Rectified linear function
RMA: Robust multi-array average
UC: Ulcerative colitis
